# Physical Activity and Thyroid Cancer Risk: A Case-Control Study in Catania (South Italy)

**DOI:** 10.3390/ijerph16081428

**Published:** 2019-04-22

**Authors:** Maria Fiore, Antonio Cristaldi, Valeriya Okatyeva, Salvatore Lo Bianco, Gea Oliveri Conti, Pietro Zuccarello, Chiara Copat, Rosario Caltabiano, Matteo Cannizzaro, Margherita Ferrante

**Affiliations:** 1 Environmental and Food Hygiene Laboratory (LIAA), Department of Medical and Surgical Sciences and Advanced Technologies “G.F. Ingrassia”, University of Catania, 95123 Catania, Italy; antonio.cristaldi81@gmail.com (A.C.); olivericonti@unict.it (G.O.C.); pietro.zuccarello@unict.it (P.Z.); ccopat@unict.it (C.C.); marfer@unict.it (M.F.); 2 General Surgery, Department of Medical and Surgical Sciences and Advanced Technologies, “G.F. Ingrassia”, 95123 Catania, Italy; okvaleria@hotmail.com (V.O.); salvo.lobianco@alice.it (S.L.B.); cannizza@unict.it (M.C.); 3 Department of Medical and Surgical Sciences and Advanced Technologies, “G.F. Ingrassia”, Section of Anatomic Pathology, 95123 Catania, Italy; rosario.caltabiano@unict.it

**Keywords:** physical activity, daily walking duration, prevention, thyroid cancer

## Abstract

Background: The health benefits of physical activity are well established, but the association between physical activity and thyroid cancer remains poorly understood. The aim of the study was to investigate the relationship between physical activity and thyroid cancer in order to determine type, frequency, and duration of exercise needed to maximize prevention. Method: Cases, diagnosed from January 2009 to July 2018, and controls were enrolled at the University Hospital “Policlinico-Vittorio Emanuele” of Catania (South Italy). Logistic regression models were used to estimate the crude and adjusted odds ratios (ORs) and their 95% confidence intervals. Results: A total of 106 cases (91.2% papillary type) and 217 controls were enrolled. Physical activity was rare in Catania (32.8%) and was not correlated to risk of total thyroid cancer (OR: 0.997; 95% CI: 0.515–1.929). Conversely, walking every day for at least 60 minutes reduced the risk of thyroid cancer (OR: 0.357; 95% CI: 0.157–0.673). Conclusions: Our study showed that daily walking duration was associated with lower risk of thyroid cancer using a case-control study. Unfortunately, the frequency of physical activity often declines with age, particularly among the elderly, thus more research on physical activity adherence is needed to determine which approaches are most effective in promoting sustained physical activity participation.

## 1. Introduction

Thyroid cancer is the most common endocrine malignancy worldwide, and it is three times more common in women compared to men [1,2,3]. Thyroid cancer incidence has grown rapidly in Italy and, as in many other countries, faster than other tumors [4,5,6,7,8,9,10]. Globally, rates of thyroid cancer are 10.2 per 100,000 in women and 3.1 per 100,000 in men. According to the World Health Organization (WHO), 93,969 new cases of thyroid cancer were estimated to have occurred in Europe in 2018. In Italy, there is a high incidence among males and females, between 7.4 and 18.9 diagnoses per 100,000 inhabitants per year [2]. The increase of the thyroid cancer incidence, observed during the last decades in most Western countries, is largely explained by the improvement of the diagnostic methodology and possibly changes in the prevalence of risk factors [11,12].

Mueller et al. (2017) estimated the number of premature deaths preventable under compliance with international exposure recommendations for physical activity, air pollution, noise, heat, and access to green spaces. In particular, Mueller’s study showed that the greatest portion of preventable deaths was attributable to an increase in physical activity. According to this evidence, several cities have been targeting reductions in air pollution and physical inactivity to improve population health [13]. The ionizing radiation and history of proliferative thyroid disease [goiter and thyroid nodules] have been established as risk factors for thyroid cancer. Other risk factors including intake of foods rich in iodine, anthropometric factors (height and weight), smoke, alcohol consumption and drugs, hormonal factors, reproductive history, and exposure to the environment are established too [14]. Whereas, the relationship between physical activity and thyroid cancer is inconclusive and minimally investigated currently [15,16,17,18,19,20,21,22,23,24,25].

Catania is an old city situated in Sicily (South Italy), and its urban planning does not have the characteristics to be considered as “Healthy and Salutogenic City” [26].

Therefore, our aim was to investigate the relationship between physical activity and thyroid cancer in Catania in order to determine the type, frequency, and duration of exercise needed to maximize prevention through a hospital-based case-control study.

## 2. Materials and Methods 

### 2.1. Materials Study Setting and Data Collection

This hospital-based case-control study was carried out in Catania (South Italy), and was revised and approved by the Ethics Committee of the University Hospital (code n. 553) “Policlinico-Vittorio Emanuele” in Catania. Informed written consent was obtained from all of the participants. 

Cases and controls were enrolled at the University Hospital “Policlinico - Vittorio Emanuele” of Catania, and all subjects were resident in Catania or neighboring municipalities. In particular, patients treated for thyroid cancer, diagnosed in the period between January 2009 and July 2018 at the Endocrine-Surgery operative unit of the hospital were eligible for the study. The Pathological Anatomy Unit of the hospital histologically confirmed all thyroid cancers. Controls were enrolled at the laboratory of clinical analysis of the hospital. The exclusion criteria for controls were subjects with diagnosed neoplasms, hormonal, or gynecological diseases.

Trained doctors, between January 2015 and July 2018, interviewed both cases and controls face-to-face. A structured questionnaire was used to collect data on demographic characteristics (age, gender, place of residence, and occupation), anthropometric parameters, reproductive and hormonal history, lifestyle (physical activity, smoking habits, alcohol consumption), exposure to radiation or chemicals, personal and family medical history. 

Information about physical activity referred to subjects’ usual pattern of activity, including sports and walking were collected using the Lifestyle Assessment Questionnaire of the Italian Health Institute [27]. In particular, we queried about the weekly frequency (only during the weekend, 2 times, every day) that participants engaged in activities of any type, and how long they usually exercised (less than 1 h, 1 h, 2 h, or more than 2 h). Daily walking duration was investigated too (less than 30 min, 30–60 min, over 60 min) [27].

Body mass index (BMI) was defined as weight (kg) divided by height (m) squared, and BMI categories were defined in accordance with the WHO classification as normal weight (18.5–24.9 kg/m^2^), overweight (25.0–29.9 kg/m^2^), and obesity (≥30.0 kg/m^2^) [28].

The subject who answered “yes” to the question “smokes habitually” (habitual smoker = who smokes at least one cigarette a day) has been considered "smoker", and the subject was considered “alcohol consumer” who reported to consume at least one glass a day of alcoholic beverages or spirits (alcohol content of up to 1.5 and 21 vol% ethanol, respectively).

### 2.2. Statistical Analysis

Descriptive statistics were performed using frequencies and percentages for qualitative variables, and mean +/- standard deviation (SD) for quantitative variables. In order to investigate the risk factors for thyroid cancer a logistic regression model was used, and results were presented as crude and adjusted odds ratio (OR) and their 95% confidence interval (95% CI). 

Statistical significance was conventionally defined as *p* < 0.05. Statistical analyses were performed using statistical software SPSS for Windows (Statistical Package for the Social Science, version 21.0; SPSS Inc., Chicago, IL, USA). 

## 3. Results

A total of 106 cases and 217 controls were included in the study. There was no significant difference in the frequency of females between cases and controls (78.1% vs. 70.0%, *p* = 0.143). The mean age of cases and controls were, respectively, 52.2 +/– 13.2 and 42.1 +/– 17.1 years, respectively. Most cases were diagnosed among women (77.7%) and in the age group <49 years (57%). The prevailing cancer was the papillary type (91.2%) (Table 1).

No significant association was found between thyroid cancer and the risk factors, profession, history of ionizing radiation exposure (diagnostic and/or therapeutic scope), and hormonal contraceptive use, between cases and controls (data not reported). Educational level, smoking status, alcohol consumption, irregular cycles, and number of pregnancies were not significantly associated with thyroid cancer (Table 2). Conversely, this study confirmed the relationship between the increase of the body mass index and the risk of thyroid cancer; briefly, subjects who were obese had a greater risk of thyroid cancer than normal weight subjects (OR_crude_: 2.966; 95% CI: 1.026–8.571). After controlling for multiple variables, the positive association persisted (OR_adj_: 1.085; 95% CI: 1.022–1.152) (Table 2).

Physical activity (yes/no) was rare in Catania (32.8% of subjects) and, after controlling for age and BMI, was unrelated to risk of total thyroid cancer (OR: 0.997; 95% CI: 0.515–1.929) (Table 3). 

We did not find an association between physical activity (yes/no), exercise duration, daily walking duration, and thyroid cancer according to histologic types (papillary vs. follicular + medullary) (data not reported). 

According with the literature, after adjustment for gender, educational level and BMI, we found an increasing thyroid cancer risk associated with age (age > 53 years: OR_adj_: 5.853, 95% CI: 2.278–15; age 39–52 years: OR_adj_: 4.820, 95% CI: 1.880–12; Reference category: <38 years), and an inverse association between age and physical activity (yes/no) (<38 years: 44.7%, 39–52 years: 31.1%, >53 years: 24.3%, *p* = 0.013). 

Exercise (OR_adj_: 0.171; 95% CI: 0.034-0.850) and daily walking duration (OR_adj_: 0.357; 95% CI: 0.157–0.673) were significantly associated with risk of total thyroid cancer. Briefly, practice an exercise for at last one hour or a daily walking for more than 60 minutes showed inverse (protective) association with total thyroid cancer. Unadjusted and adjusted odds ratios were very similar (Table 3).

## 4. Discussion

This case-control study is the first to focus on the relationship between physical activity and thyroid cancer in the Catania population and Italy. The most important finding of our study was the decreased thyroid cancer risk associated with both the “exercise duration” (OR: 0.171; 95% CI: 0.034–0.850) and the “daily walking duration” (OR: 0.357; 95% CI: 0.157–0.673).

Few studies have investigated the relationship between physical activity and thyroid cancer, and their results are contrasting [15,19,20,21,22,23,24]. Therefore, the relationship between physical activity and thyroid cancer remains unclear. Rossing et al. in a case-control study among women reported an OR of papillary thyroid cancer of 0.76 (95% CI: 0.59–0.98) for regular exercise during the two years before diagnosis compared to no regular exercise [15]. We did not find an association between physical activity (yes/no), exercise duration, daily walking duration and thyroid cancer according to histologic types (papillary vs. follicular + medullary) probably because the analysis was based on only nine (five follicular and four medullary) exposed cases. A pooled analysis of two case-control studies found an overall significant but a rather weak negative association between physical activity and thyroid cancer risk. This association is higher for a more frequent practice rather than for a longer duration of practice [29]. Our results have shown a protective effect of the physical activity (yes/no) carried out at least twice a week with a duration of not less than one hour. Furthermore, our results highlight the protective effect of daily walking for at least 30 minutes. Walking is one of the physical activities that does not require special training or equipment and can be integrated easily into daily life [30,31], therefore this result should be taken into account within the organization of the preventive interventions because the risk of thyroid cancer increases with age, and the elderly subjects practice less physical activity, including walking.

Many international institutions recommend that adults engage in moderate-intensity physical activity for at least 30 minutes a day for five days a week, or exercise at a high intensity for at least 20–30 minutes, three or more days a week [32]. Despite the protective effect of physical activity, 1 in 4 adults and 3 in 4 adolescents (aged 11–17 years), worldwide, do not currently meet the global recommendations for physical activity set by the WHO as countries develop economically, and the levels of inactivity were increased. In some countries, the levels of inactivity can be as high as 70%, due to changing patterns of transportation, increased use of technology and urbanization. The global cost of physical inactivity is estimated to be INT$ 54 billion per year in direct health care, in 2013, with an additional INT$ 14 billion attributable to lost productivity [33].

Physical activity has been hypothesized to influence cancer risk through several mechanisms [34]. DNA repair and hormonal pathways may be some of the several mechanisms whereby the cancers risk might decrease [17], but these mechanisms are not yet entirely clear. Exercise plays a role in mediating the effects of chronic inflammation, reducing inflammatory markers such as C-reactive protein (CRP), tumor necrosis factor alpha, and various types of interleukin (IL), including IL6, in people with and without cancer [12,35,36,37,38]. Furthermore, the protective effects of exercise have been attributed to the creation of an anti-inflammatory environment through increasing anti-inflammatory cytokines such as IL-1ra and IL10 in healthy people [21]. Another hypothesis is that the practice of physical activity may influence thyroid cancers risk by reducing obesity [21]. 

This study has some limitations. Sample size may be the main limitation. Others limitations of our study involve imprecise estimation of physical activity because of the self-reported nature of our data [39], but, validation studies comparing physical activity assessments similar to those used in our case-control study with referent methods indicate that the reliability and validity of our instrument is comparable to self-reported measures used in similar studies [40]. We did not discuss race/ethnicity because our sample consisted entirely of Caucasians, but we controlled for the other numerous potential confounding variables, including age, gender, history of radiation exposure, education level, smoking status, alcohol use, and oral contraceptive use among women. 

## 5. Conclusions 

Our study shows that daily walking for at least 30 minutes might have a protective effect on thyroid cancer risk using a case-control study performed in the University Hospital “Policlinico-Vittorio Emanuele” of Catania. This study confirms the importance of a non-sedentary lifestyle for the prevention of thyroid cancer, but the frequency of physical activity often declines with age, particularly among the elderly. Thus, more research on physical activity adherence is needed to determine which approaches are most effective in promoting sustained physical activity participation.

Moreover, globalization and urbanization, together with the aging of the population, interact with the determinants of social, cultural, and economic health, exposing people to behavioural risk factors such as physical inactivity. Therefore, the cities should be redesigned to encourage physical activity [26].

## Figures and Tables

**Table 1:** Characteristics of cases.

Variables	n	%
**Gender**		
Female	80	77.7
Male	23	22.3
**Age at diagnosis**		
<49	57	57.0
50–69	38	38.0
>70	5	5.0
**Histology**		
Papillary	93	91.2
Follicular	5	4.9
Medullary	4	3.9

**Table 2:** Odds ratio (ORs) and 95% Confidence Interval (CI) of thyroid cancer risk factors.

Variables	Case n (%)	Control n (%)	Crude OR (95% CI)	Adjusted OR ^1^ (95% CI)
**Education level**				
Elementary school	17(16.3)	13 (6.0)	*	*
Middle school	31 (29.8)	53 (24.5)	0.447 (0.192–1.044)	0.397 (0.066–2.371)
High school	40 (38.5)	111 (51.4)	0.289 (0.129–0.647)	0.271 0.037–1.964)
University	16 (15.4)	39 (18.1)	0.314 (0.124–0.793)	0.667 (0.074–6.013)
**BMI ^2^**	22.2+/–4.7	20+/–3.8	**1.120** **(1.058–1.185)**	**1.118** **(1.019–1.225)**
**BMI ^2^**				
18.5–24.9	58 (69.9)	105 (82.0)	*	*
25.0–29.9	15 (18.1)	17 (13.3)	1.570 (0.731–3.371)	3.728 (0.606–22)
≥30.0	10 (12.0)	6 (4.7)	**2.966** **(1.026–8.571)**	**7.581** **(1.295–44)**
**Smoking status**				
Never smoker	80 (77.7)	155 (71.4)	*	*
Current smoker	23 (22.3)	62 (28.6)	0.741 (0.431–1.274)	0.281 (0.063–1.253)
**Alcohol consumption**				
No	74 (72.5)	176 (81.1)	*	*
Yes	28 (27.5)	41 (18.9)	1.660 (0.961–2.868)	2.081 (0.534–8.107)
**Irregular cycles**				
No	39 (78.0)	67 (83.8)	*	*
Yes	11 (22.0)	13 (16.3)	1.383 (0.567–3.374)	2.090 (0.421–10)
**Number of pregnancies**				
Nullipara	8 (10.7)	15 (19.2)	*	*
1	9 (12.0)	13 (16.7)	1.154 (0.353–3.776)	1.308 (0.103–16)
2	39 (52.0)	33 (42.3)	1.970 (0.764–5.081)	1.063 (0.193–5.868)
3 and more	19 (25.3)	17 (21.8)	1.863 (0.649–5.345)	0.452 (0.050–4.074)

^1^ Each odds ratio is adjusted for gender, age, and for all other variables in the table. * Reference category. ^2^ BMI: Body mass index; weight (kg)/height (m^2^). Bold indicates ORs statistically significant.

**Table 3:** OR of thyroid cancer by aspect of physical activity.

	Case	Control	Crude OR (95% CI)	Adjusted OR ^1^ (95% CI)
**Practice regular physical activity**				
Yes	26 (25.0)	80 (36.9)	*	*
No	78 (75.0)	137 (63.1)	**1.797** **(1.067–3.026)**	0.997 (0.515–1.929)
**Weekly frequency**				
Only during weekend	5 (20.0)	9 (11.4)	*	*
2 times	16 (64.0)	19 (74.7)	0.488 (0.143–1.661)	0.352 (0.080–1.556)
Every day	4 (16.0)	11 (13.9)	0.655 (0.134–3.186)	0.608 (0.083–4.437)
**Exercise duration**				
Less than 1 h	5 (20.0)	3 (3.8)	*	*
1 h	12 (48.0)	52 (62.8)	**0.138** **(0.029–0.661)**	**0.171** **(0.034–0.850)**
2 h	7 (28.0)	18 (22.8)	0.233 (0.044–1.248)	0.480 (0.052–4.425)
Over 2 h	1 (4.0)	6 (7,6)	0.100 (0.008–1.288)	0.145 (0.008–2.719)
**Daily walking duration**				
Less than 30 min	60 (60.0)	90 (41.9)	*	*
30–60 min	30 (30.0)	75 (34.9)	0.590 (0.346–1.006)	**0.563** **(0.322–0.984)**
Over 60 min	10 (10.0)	50 (23.3)	**0.325** **(0.157–0.673)**	**0.357** **(0.157–0.673)**

^1^ Each odds ratio is adjusted for age and BMI. * Reference category. Bold indicates ORs statistically significant.
